# A multi-omics approach reveals function of Secretory Carrier-Associated Membrane Proteins in wood formation of​ ​​*Populus​*​ ​trees

**DOI:** 10.1186/s12864-017-4411-1

**Published:** 2018-01-03

**Authors:** Ogonna Obudulu, Niklas Mähler, Tomas Skotare, Joakim Bygdell, Ilka N. Abreu, Maria Ahnlund, Madhavi Latha Gandla, Anna Petterle, Thomas Moritz, Torgeir R. Hvidsten, Leif J. Jönsson, Gunnar Wingsle, Johan Trygg, Hannele Tuominen

**Affiliations:** 1 Umeå Plant Science Centre, Department of Plant Physiology, Umeå University, 90187 Umeå, Sweden; 2 Umeå Plant Science Centre, Department of Forest Genetics and Plant Physiology, Swedish University of Agricultural Sciences, 90183 Umeå, Sweden; 3 Department of Chemistry, Umeå University, 90187 Umeå, Sweden; 4 Computational life science cluster (CLiC), Department of Chemistry, Umeå University, Umeå, Sweden; 5 Faculty of Chemistry, Biotechnology and Food Science, Norwegian, University of Life Sciences, 1432 Ås, Norway; 6 Present address: Department of Microbiology and Immunology, Institute of Biomedicine, University of Gothenburg, 40530 Gothenburg, Sweden

**Keywords:** Secretory Carrier-Associated Membrane Protein (SCAMP), *Populus*, Wood chemistry, Wood density, Biomass, Bioprocessing, Cork, Multi-omics

## Abstract

**Background:**

Secretory Carrier-Associated Membrane Proteins (SCAMPs) are highly conserved 32–38 kDa proteins that are involved in membrane trafficking. A systems approach was taken to elucidate function of SCAMPs in wood formation of *Populus* trees. Phenotypic and multi-omics analyses were performed in woody tissues of transgenic *Populus* trees carrying an RNAi construct for *Populus tremula x tremuloides SCAMP3* (*PttSCAMP3;* Potri.019G104000).

**Results:**

The woody tissues of the transgenic trees displayed increased amounts of both polysaccharides and lignin oligomers, indicating increased deposition of both the carbohydrate and lignin components of the secondary cell walls. This coincided with a tendency towards increased wood density as well as significantly increased thickness of the suberized cork in the transgenic lines. Multivariate OnPLS (orthogonal projections to latent structures) modeling of five different omics datasets (the transcriptome, proteome, GC-MS metabolome, LC-MS metabolome and pyrolysis-GC/MS metabolome) collected from the secondary xylem tissues of the stem revealed systemic variation in the different variables in the transgenic lines, including changes that correlated with the changes in the secondary cell wall composition. The OnPLS model also identified a rather large number of proteins that were more abundant in the transgenic lines than in the wild type. Several of these were related to secretion and/or endocytosis as well as both primary and secondary cell wall biosynthesis.

**Conclusions:**

*Populus* SCAMP proteins were shown to influence accumulation of secondary cell wall components, including polysaccharides and phenolic compounds, in the woody tissues of *Populus* tree stems. Our multi-omics analyses combined with the OnPLS modelling suggest that this function is mediated by changes in membrane trafficking to fine-tune the abundance of cell wall precursors and/or proteins involved in cell wall biosynthesis and transport. The data provides a multi-level source of information for future studies on the function of the SCAMP proteins in plant stem tissues.

**Electronic supplementary material:**

The online version of this article (10.1186/s12864-017-4411-1) contains supplementary material, which is available to authorized users.

## Background

Forest trees are an important source of renewable products such as biofuels and bioenergy. They are highly efficient in incorporating atmospheric carbon into the tree trunk, and increased forest stocks have recently been identified by the Intergovernmental Panel on Climate Change as, possibly, the most efficient way to combat further increases in atmospheric CO_2_ [1]. Forest stocks can be increased by increasing carbon flow into the secondary cell walls in the woody tissues of the stem. Several different approaches have been taken to modify biosynthesis of the individual secondary cell wall components for the purpose of increased biomass production. One of them is reducing lignin content by genetic engineering, in natural variants or in forest tree hybrids, which has in some cases been shown to increase the growth of forest trees [2–4], but in other cases to impair tree growth and also the water transport capacity of the trees [5–8]. Only a few reports exist on targeted modification or natural variants of genes that promote cellulose or hemicellulose biosynthesis. Overexpression of a sucrose synthase induced an increase in cellulose biosynthesis and wood density without interfering with growth of hybrid poplar trees [9]. Overexpression of the hemicellulose catabolic xyloglucanase also increased cellulose content and density of the wood in white poplar [10], but reduced tree growth in long-term cultivation [11]. Due to the imminent risk for growth penalty when modifying secondary cell wall biosynthesis, it is important to find additional genetic engineering strategies to improve biomass production of forest trees in a manner that does not have adverse effects on the growth of the trees.

Multi-omics approaches, including simultaneous profiling of the transcriptome, proteome and the metabolome, can be helpful when trying to improve complex processes such as growth and biomass production [12, 13]. Such multi-omics approaches have so far not been extensively utilized in forest trees [14] but have proven instrumental in other contexts, for example in creating strategies to increase carotenoid biosynthesis in maize kernels [15] or secondary metabolite accumulation in grape berries [16]. In addition to being quite expensive, a multidisciplinary approach can be challenging due to difficulties encountered when interpreting the multilayered datasets. A good solution for the latter problem is to employ multivariate methods such as OnPLS (orthogonal projections to latent structures) [17, 18] which can handle noisy, multicollinear datasets with many more variables than samples and identify significant variation in several datasets at the same time [14].

Here we took a multidisciplinary approach, combined with the most recent development of the OnPLS method, to analyze data from a set of transgenic *Populus* trees that had been identified among a large number of transgenic trees as having increased biomass production rates. The transgenic lines carried an RNAi construct for the *PttSCAMP3* (*Populus* *tremula x* *tremuloides* **S**ecretory **Ca**rrier-**A**ssociated **M**embrane **P**rotein3) gene. The SCAMPs are highly conserved 32–38 kDa proteins that are localized in the endomembranes and the plasmamembrane and that, in animals, seem to be mainly involved in exocytosis in specialized secretory cells but also endocytosis and multivesicular endosome biogenesis [19]. In plants the function is unclear, although a role in lily pollen tube growth has been suggested [20]. *Arabidopsis* mutants in the SCAMP genes have not exhibited any obvious phenotypes [19]. Our analysis identified changes in wood chemistry, wood density and enzyme-catalyzed cell wall digestibility of the *PttSCAMP3* RNAi lines. The underlying mechanisms were elucidated by analyses of the transcriptomes, proteomes and metabolomes combined with the OnPLS modelling. These results revealed not only a critical function for the SCAMP-dependent pathway in wood chemistry but also provided a systems-level interpretation of biological responses and pathways controlled by the SCAMP proteins in the woody tissues of *Populus*.

## Methods

### Transformations, growth conditions and tissue sampling

An RNAi construct for *PttSCAMP3* was created by amplifying a fragment from a cDNA clone “EST G066P24” corresponding to Potri.019G104000 (SCAMP3) with the forward primer GGGGACCACTTTGTACAAGAAAGCTGGGTCTGGAGGCTATGTTATGTGGTATCG and reverse primer GGGGACAAGTTTGTACAAAAAAGCAGGCTGACACTGAGGAGTGATTCAACGC, followed by recombination into pDONOR201 and further into pK7GWIWG2(I), resulting in a hairpin structure of two inverted *PttSCAMP3* fragments under the control of the Cauliflower Mosaic Virus 35S promoter. The resulting vector was transformed into hybrid aspen (*Populus tremula x tremuloides*) clone T89 according to [21]. A large number of transgenic lines were regenerated, of which three were selected for detailed analyses.

Material from wild type (WT) and transgenic trees was amplified in vitro, and 33 wild type trees and five trees for each of the transgenic lines were grown in the greenhouse in K-soil (Hasselfors Light peat with sand and clay, Hasselfors Garden AB, Örebro, Sweden) with an 18 h day length, day/night temperature of 20/15 °C and relative humidity of 50–70%. The trees were grown in a random order, rotated on a weekly basis, and fertilized once a week after 3 weeks of growth with Horto NPK 7–1-5 Rika-S (Weibulls, Åby, Sweden). After 2 months of growth, the stem height and diameter of the stem at the base of each tree was measured. Next day trees were harvested and the bottom part of the stem, excluding the lowermost 10 cm, was collected for the various analyses. A seven-cm piece from the stem (10–17 cm from the base of the stem) was collected for the analyses of the metabolome, proteome and transcriptome, flash frozen with liquid nitrogen and stored at −80 °C. A three-cm piece (33–36 cm from the base) was collected for anatomical inspection and placed in FAA (5% formaldehyde, 5% acetic acid, 50% ethanol) and stored at 4 °C. A further ten-cm piece (36–46 cm from the base) was cut and used for density measurements, pyrolysis-gas chromatography/mass spectrometry (Py-GC/MS), monosaccharide analyses and analytical enzymatic saccharification, and stored at −20 °C. Stem dry weight was measured (together with a repetition of density measurements) from a separate experiment where whole stems were dried and weighed.

### Analyses of the transcriptome, proteome and metabolome

Seven WT trees and five trees for each of the transgenic lines were selected for transcriptome, proteome and metabolome analyses. The wild type trees were selected on the basis of a PCA score plot to cover the variation in growth across the whole population of the wild type trees. The seven-cm piece that was collected from the stem was peeled, and the living part of the xylem was scraped away with a scalpel and flash frozen in liquid nitrogen as described in [22]. All samples were ground to a fine powder in a mortar cooled with liquid nitrogen and stored at -80 °C.

#### The analysis of the transcriptome

Total RNA was extracted using a RNeasy mini kit (Qiagen) supplemented with the RNAse-free DNAse set (Qiagen) and RNeasy MinElute cleanup kit (Qiagen). The protocol was based on the standard in-house protocol and on the manufacturer’s instructions. RNA integrity was assessed by gel electrophoresis on agarose gel (staining with gel-red) and using a Bioanalyser 2100 (Agilent Technologies, Waldbronn, Germany). RNA sequencing (Illumina, 100 bp paired-end reads) was performed at the Beijing Genome Institute (China), and the analysis was carried out according to their standard procedure. Raw data were pre-processed and aligned using the RNA-Seq pipeline described in [23]. In short, reads were filtered for ribosomal RNA, trimmed and aligned to version 3 of the *Populus trichocarpa* reference genome [24–26] with STAR [27]. The number of reads aligning to annotated gene models was determined using HTSeq [28]. Read counts were normalized with a variance stabilizing transformation (VST) implemented in the R-package DESeq2 [29]. These gene expression values were used in further downstream analyses.

Quantitative PCR (qPCR) analysis was run for RNA samples from three replicate trees per genotype after a DNAse treatment with DNA-free TM kit (Ambion), cDNA synthesis by iScript cDNA synthesis kit (Bio-Rad) and qPCR with LightCycler® 480 II (Roche) to analyse expression of *PttSCAMP3* using primers GGAGGCTATGTTATGTGGTATCGC and CAGAGCACTATCTGTCCTCATTGC. A cyclophilin gene (Potri.004G168800) [30] was chosen as a reference gene using GeNorm software as described earlier [31], and amplified with primers GGCTAATTTTGCCGATGAGA and ACGTCCATCCCTTCAACAAC.

#### The analysis of the proteome

Total proteins were extracted from 20 mg of frozen stem tissue powder as described earlier [32]. The samples were run on a Synapt™ G2 HDMS mass spectrometer (Waters, UK) equipped with a nanoflow electrospray ionization interface according to [33]. Protein identification and peptide quantification was described earlier [14].

#### The analysis of the metabolome with GC-MS and LC-MS

For gas chromatography-mass spectrometry (GC-MS), metabolites were extracted and their profiles analyzed using an Agilent 6890 GC coupled to a Pegasus III time of flight MS, as described in [34]. The generated files were processed and the metabolites identified as described in [35].

For ultra high performance liquid chromatography-mass spectrometry (UHPLC-MS) analysis, one mL of extraction buffer (20/20/60 *v*/v chloroform:water:methanol) including the internal standards Reserpine (Sigma), Sulfadimethoxine (Fluka), Leucine-Enkephalin (Fluka) and Val-Tyr-Val (Bachem) was added to 9–12 mg of the plant material. The sample was shaken with a tungsten bead in a mixer mill at 30 Hz for 3 min, the bead was removed and the sample was centrifuged at +4 °C, 14,000 rpm, for 10 min. Then, 200 μL of supernatant were transferred to a micro vial and the solvents were evaporated. Before analysis, the sample was re-suspended in 10 + 10 μL methanol and water (with 0.1% formic acid). The chromatographic separation was performed on an Agilent 1290 Infinity UHPLC-system (Agilent Technologies, Waldbronn, Germany). Two μL of re-suspended aliquots of extracted plant sample were injected onto a 2.1 × 100 mm, 1.7 μm Kinetex C18 column (Phenomenex, Torrace, USA) held at 40 °C. The gradient elution buffers were A (H_2_O, 0.1% formic acid) and B (acetonitrile, 0.1% formic acid), and the flow-rate was 0.5 mL min^−1^. The compounds were eluted with a linear gradient consisting of 1–20% B over 0–4 min, 20–40% B over 4–6 min, 40–95% B over 6–9 min, the composition was held at 95% B for 4.5 min, and returned to 1% B at 14.50 min, the composition was kept at 1% B for a further 4.5 min before the next injection. The diode array detector was set to scan the interval 190–640 nm with a step length of 2 nm and a slit width of 2 nm. The compounds were detected with an Agilent 6540 Q-TOF mass spectrometer equipped with a jet stream electrospray ion source operating in negative ion mode. The settings were kept identical between the modes, with the exception of the capillary voltage. A reference interface was connected for accurate mass measurements; the reference ions purine (4 μM) and HP-0921 (Hexakis(1H, 1H, 3H–tetrafluoropropoxy phosphazine) (1 μM), both purchased from Agilent Technologies (Santa Clara, CA, USA), were infused directly into the MS at a flow rate of 0.05 mL min^−1^ for internal calibration, and their monitored ions were m/z 119.03632 and m/z 966.000725 for negative mode, respectively. The gas temperature was set to 300 °C, the drying gas flow to 8 L min^−1^ and the nebulizer pressure to 40 psig. The sheath gas temp was set to 350 °C and the sheath gas flow to 11 L min^−1^. The capillary voltage was set to 4000 V. The nozzle voltage was 0 V. The fragmentor voltage was 100 V, the skimmer 45 V and the OCT 1 RF Vpp 750 V. The collision energy was set to 0 V. The m/z range was 70–1700, and data were collected in centroid mode with an acquisition rate of 4 scans s^−1^ (1974 transients/spectrum). Mass Feature Extraction (MFE) from the data acquired was performed using the MassHunter™ Qualitative Analysis software package, version B05.00 (Agilent Technologies Inc., Santa Clara, CA, USA). Extracted features were aligned and matched between samples using Mass Profiler Professional™ 12.5 (Agilent Technologies Inc., Santa Clara, CA, USA).

The metabolite annotation was done by manual interpretation of the fragments with high mass accuracy or by searches in an *in house* database. For critical samples, extracts from transgenic and wild-type plants were re-analyzed by Liquid Chromatography Quadrupole Time-of-Flight Mass Spectrometry (LC-Qtof) targeted MS/MS approach using the same chromatographic and mass spectrometry conditions as described above, with collision energy set up from 10 to 40 V. The metabolomic extracts were also re-analyzed by a lipidomic approach [36] to improve annotations of metabolites with long retention times.

### Wood chemical analyses

The ten-cm stem piece (36–46 cm from the base) that was collected for the chemical analyses was freeze-dried for about 48–72 h, cut into small pieces (1 cm long × 1 mm diameter) and ground into a rough powder using a centrifugal mill (Retsch ZM 200, Haan, Germany). For analytical enzymatic saccharification and monosaccharide analysis by acid hydrolysis, the rough powder was sieved to a particle size between 0.1 and 0.5 mm using an analytical sieve shaker AS 200 (Retsch). For pyrolysis-GC/MS, the rough powder was further ground into a fine powder using a ball mill (Retsch MM400) for 150 s at 30 Hz, as described previously [37].

#### Pyrolysis-gas chromatography/mass spectrometry (Py-GC/MS)

About 50 μg of fine wood powder, weighed using a micro balance (XP6U, Mettler Toledo, USA) was analyzed by a pyrolyzer (PY-2020iD and AS-1020E, Frontier Lab, Japan) connected to a GC-MS (7890A/5975C; Agilent Technologies AB, Sweden), as described previously [38]. All 33 wild type trees and 5 replicate trees from the RNAi lines were analyzed.

#### Monosaccharide analysis by acid hydrolysis

Sieved rough wood powder from each of the transgenic lines (with three technical replicates, each of which containing equal amounts of wood powder pooled from five biological replicates of the transgenic trees and from five pools of wild type trees) was used to determine total monosaccharide content after acid hydrolysis. Dry wood powder (100 mg, after moisture analysis using Mettler Toledo HG63, Switzerland) was hydrolyzed with sulfuric acid (3 ml, 72% (*w*/w)] for 1 h at 30 °C). The reaction mixture was then diluted to 4% sulfuric acid using deionized water and autoclaved for 1 h at 120 °C. After centrifugation (14,000 *g* for 20 min), the supernatant was collected and analyzed for monosaccharide sugars using high-performance anion-exchange chromatography (HPAEC), as described previously [37].

### Analytical enzymatic saccharification

Sieved wood powder (50 mg) from each sample (each transgenic line containing five biological replicates and the wild type containing five pools of biological replicates, each pool consisting of equal amounts of wood from 4 to 6 wild type trees) was subjected to enzymatic hydrolysis, with and without prior thermochemical pretreatment. The thermochemical pretreatment was performed as previously described [37] by impregnation with 1% (w/w) sulfuric acid and treatment at 165 °C for 10 min using an Initiator single-mode microwave instrument (Biotage, Uppsala, Sweden). The pretreated wood was divided into a liquid phase, referred to as pretreatment liquid, and a solid phase consisting mainly of cellulose and lignin, which, after washing, was used as a substrate for cellulolytic enzymes. Analytical enzymatic saccharification of non-pretreated and pretreated wood was described earlier [37]. Briefly, milled and sieved wood or the solid phase after the pretreatment was digested enzymatically for 72 h at 45 °C by addition of commercially available liquid preparations of Celluclast 1.5 L (Sigma-Aldrich) and Novozyme 188 (Sigma-Aldrich). Samples for rapid glucose analysis using a glucometer [37] were withdrawn after 2 h for determination of the glucose production rate (GPR). The monosaccharide contents of samples taken at the end of the reaction, after 72 h, were analyzed using High-performance anion-exchange chromatography (HPAEC) for determination of the sugar yields, as previously described [37]. The monosaccharide contents of the pretreatment liquid were also analyzed using HPAEC.

### Data integration and statistical analysis by OnPLS

Data acquired from five platforms (transcriptomics, proteomics, GC-MS, LC-MS and Py-GC/MS) were integrated by OnPLS. The data sets from the three different transgenic lines were combined into one dataset and preprocessed as described earlier [39]. In short, the datasets from the transgenic lines were normalized relative to WT by subtracting the average WT value from the value of each data point and dividing by the standard deviation (SD) of the wild type. Model significance was determined using the so-called leave-one-out cross-validation [40], and implemented here using p(CORR) value as a measure of the significance of the variation [41]. An arbitrary cutoff value |p(CORR)| > 0.5 was applied here to identify statistically significant variation between the transgenic lines and the wild type similar to the approaches taken by Tulipani et al. [42] and Llorach et al. [43–45].

## Results

### Description of the SCAMP gene family in *Populus*

**Fig. 1:**
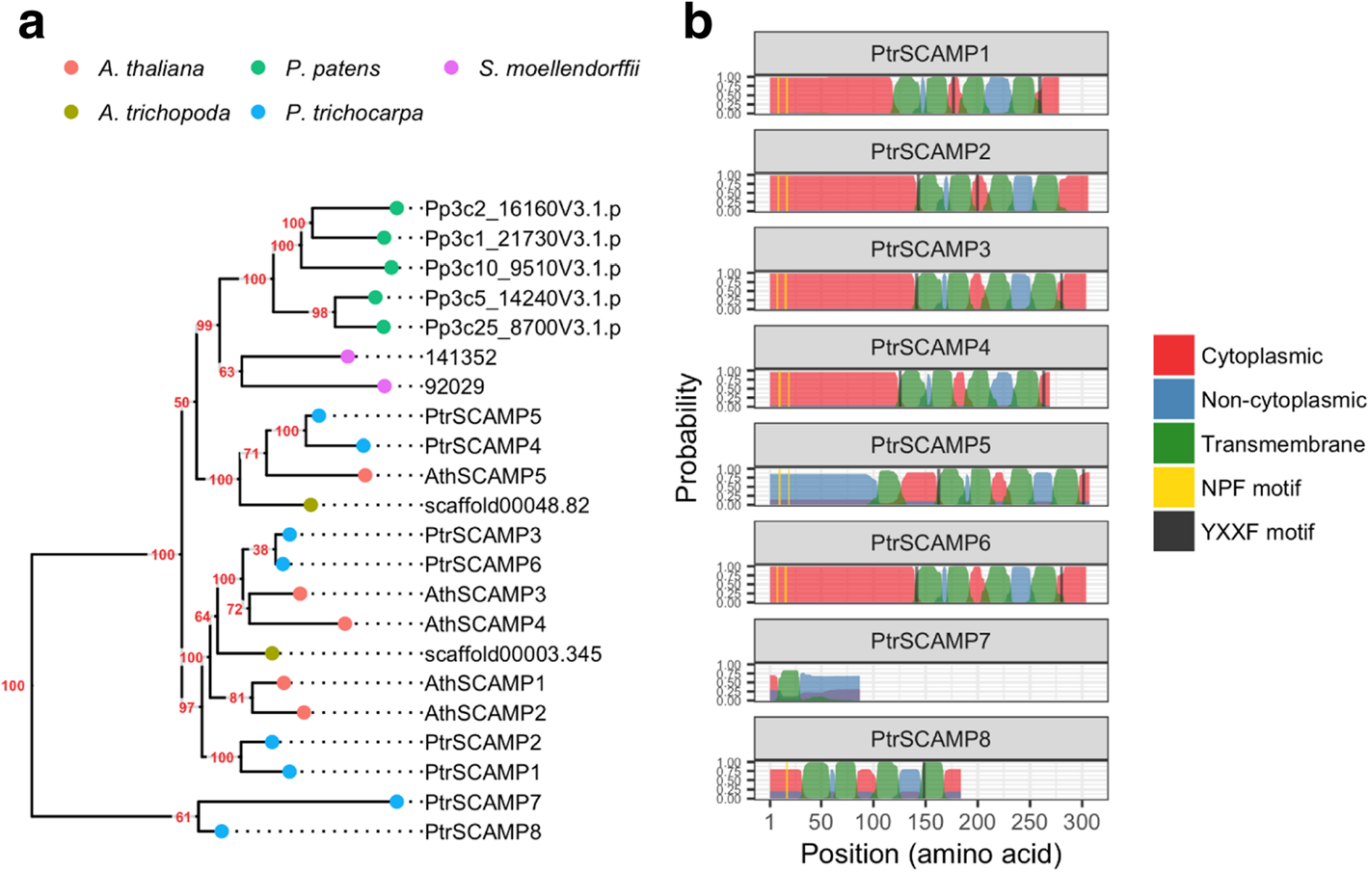
Phylogenetic analysis of SCAMP gene families and transmembrane topology analysis of *P. trichocarpa* SCAMP proteins. **a** The gene families were retrieved from the Joint Genome Institute (JGI) (http://genome.jgi.doe.gov/). The SCAMP genes are evm_27.model.AmTr_v1.0_scaffold00003.345 and evm_27.model.AmTr_v1.0_scaffold00048.82 in *Amborella trichopoda*, AT1G61250 (AthSCAMP1), AT1G11180 (AthSCAMP2) AT2G20840 (AthSCAMP3), AT1G03550 (AthSCAMP4) and AT1G32050 (AthSCAMP5) in *Arabidopsis thaliana*, Phpat.001G082700 (Pp3c1_21730V3.1.p), Phpat.002G068200 (Pp3c2_16160V3.1.p), Phpat.025G032600 (Pp3c25_8700V3.1.p), Phpat.010G037000 (Pp3c10_9510V3.1.p) and Phpat.005G053500 (Pp3c5_14240V3.1.p) in *Physcomitrella patens*, Potri.004G036600 (PtrSCAMP1), Potri.011G045100 (PtrSCAMP2), Potri.019G104000 (PtrSCAMP3), Potri.001G134100 (PtrSCAMP4), Potri.003G099300 (PtrSCAMP5), Potri.013G144700 (PtrSCAMP6), Potri.011G045200 (PtrSCAMP7) and Potri.004G036700 (PtrSCAMP8) in *Populus trichocarpa*, and 141,352 and 92,029 in *Selaginella moellendorffii*. The evolutionary history was inferred using the WAG substitution model [58]. Evolutionary analyses were conducted in R (https://www.R-project.org/) using the phangorn package (v2.2.0) and visualised using the ggtree extension for ggplot2. Numbers are bootstrap support values based on 1000 runs. **b** Transmembrane topology prediction was performed using Phobius and visualised using ggplot2. The NPF motif and the tyrosine sorting motif YXXF were identified using the regular expressions NPF and YXXF, respectively, in R (https://www.R-project.org/)

The SCAMP genes encode highly conserved proteins which normally form small gene families. *Populus* genus has eight SCAMP gene family members. Phylogenetic analysis was performed to investigate *Populus trichocarpa* SCAMP (PtrSCAMP) sequence similarity with two angiosperm species, *Arabidopsis thaliana* and *Amborella trichopoda*, the bryophyte *Physcomitrella patens* and the lycophyte *Selaginella moellendorffii*. The analysis revealed two clusters containing the SCAMP sequences from the three angiosperm species (Fig. 1a), similar to what was described previously [19]. A third cluster contained sequences from *Physcomitrella patens* and *Selaginella moellendorffii*. Two *Populus trichocarpa* sequences (PtrSCAMP7 and PtrSCAMP8) were separate from these three clusters, suggesting they might be functionally divergent. It has previously been reported that most plant SCAMP proteins have a cytoplasmic N-terminal with NPF motifs, four transmembrane domains, and a cytoplasmic C-terminal containing the tyrosine sorting motif YXXF [19, 46]. The domain structure of the *Populus* SCAMP gene family was analysed here, showing that PtrSCAMP1–4, PtrSCAMP6, and PtrSCAMP8 have cytoplasmic tails and four transmembrane domains each, PtrSCAMP1 and PtrSCAMP3–6 have the C-terminal YXXF motif and PtrSCAMP1–6 have two N-terminal NPF motifs (Fig. 1b).

**Fig. 2:**
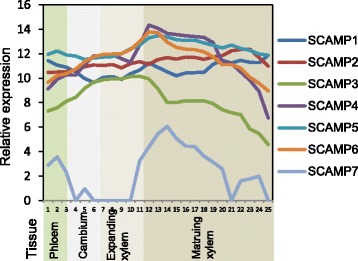
Expression profile of the *Populus* SCAMP gene family members in *Populus* stem. The data were retrieved from the AspWood database (http://aspwood.popgenie.org) where relative expression (relative to the number of RNA-Seq reads in the sample and VST normalized) is shown for aspen stem samples which consist primarily of phloem, cambium, expanding xylem and maturing xylem. Data is shown for tree 1. Similar results were obtained for three additional replicate trees in the AspWood database

Next, we analyzed expression of the *Populus* SCAMP gene family members in the woody tissues of the stem using the AspWood gene expression database (http://aspwood.popgenie.org) which contains high-resolution RNA sequencing data from the different tissue types of the aspen (*Populus tremula*) stem [47]. The analysis revealed that all *Populus tremula SCAMPs* (PtSCAMP) except for *PtSCAMP8* are expressed in the stem (Fig. 2). They all have quite similar expression patterns, with a peak of expression right at the beginning of the maturation zone which is the location for the initiation of secondary cell wall formation. Notably, *PtSCAMP3* shows a peak of expression in the xylem a little earlier than the others. *PtSCAMP7*, which was divergent from the others in the phylogenetic analysis, exhibited similar, although somewhat lower, expression than the other *Populus SCAMP* genes. On the basis of this, we can conclude that there are seven *SCAMP* genes in *Populus* that are expressed in the woody tissues in a manner suggestive of roles during xylem expansion and/or initiation of xylem maturation.

Transgenic lines modified in the expression of *Populus SCAMP3* were analyzed in this study, and we therefore used the AspWood database to find genes that were co-expressed with *PtSCAMP3* in the aspen woody tissues. Interestingly, several nucleotide-diphospho-sugar transferases, as well as genes related to cell wall biosynthesis, were among the most co-expressed genes (Additional file 1).

### Suppression of two *SCAMP* genes in transgenic *Populus* trees results in increased accumulation of secondary cell wall components in the stem

**Fig. 3:**
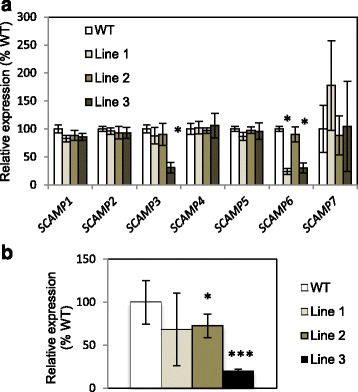
Expression of the *PttSCAMP* genes in the wild type and the three *PttSCAMP3* RNAi lines. **a** Relative expression (as a percentage of the WT) is shown for differentiating xylem samples scraped from the base of two-month-old trees. The expression is based on gene expression values in the RNA sequencing datasets, where read counts were normalized for the RNAseq library size. Asterisks indicate significant difference from the wild type at P(Benjamini-Hochberg adjusted) < 0.05 according to the R-package DESeq2. Vertical bars indicate ± SD. *n* = 5. **b** Relative expression of *PttSCAMP3* (as a percentage of the WT) by qPCR analysis. Asterisks indicate significant difference from the wild type at *P* < 0.05 (*) or *P* < 0.001 (***) according to Welch corrected t-test. Vertical bars indicate ± SD. Three biological replicates were analyzed in three technical replicates each

Functional analyses were performed in transgenic *Populus tremula x tremuloides* (*Ptt*) trees carrying an RNAi construct for *PttSCAMP3*. RNA sequencing of three transgenic lines showed a 2–69% decrease in the expression of *PttSCAMP3*. *PttSCAMP3* is paralogous with *PttSCAMP6*, and the RNAi construct resulted in 9–75% decrease in the expression of *PttSCAMP6* as well, while the expression of the other *PttSCAMP* genes were only slightly changed in the different lines (Fig. 3a). The RNAi lines therefore reflect the combined function of *PttSCAMP3* and *PttSCAMP6* in line three and the function of *PttSCAMP6* in line 1. Verification of the RNAseq results by qPCR revealed suppression of *PttSCAMP3* also in line 2 (Fig. 3b).

**Fig. 4:**
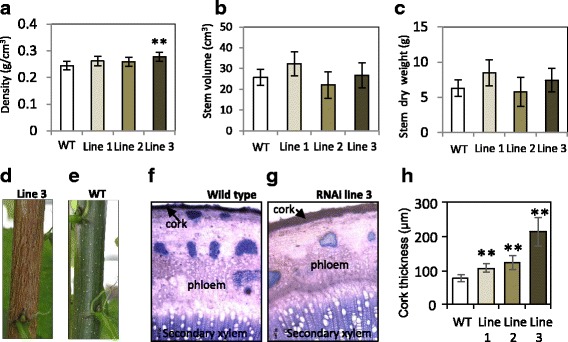
Phenotypic analysis of the wild type and the *PttSCAMP3* RNAi lines. Wood density at the base of the stem (**a**), stem volume (**b**) and stem dry weight (**c**) in two-months-old greenhouse grown trees. Representative images of the stems of six-month**-**old wild type (**d**) and *PttSCAMP3* RNAi line 3 (**e**) trees. **f-g** Light microscopy images of toluidine blue stained transverse sections taken from the base of the stems of two-months-old greenhouse grown wild type (**f**) and *PttSCAMP3* RNAi line 3 (**g**) trees. H. Thickness of the cork in the wild type and the *PttSCAMP3* RNAi lines at the base of the stems of two-months-old greenhouse grown trees. Asterisks indicate significant differences from the wild type at *P* < 0.01 (**) according to a Welch-corrected t-test. The volume of the stem is estimated with the formula volume = π · radius2 · height / 3. Vertical bars indicate ± SD. *n* = 5

Detailed phenotypic analysis of 2-month-old, greenhouse-grown trees revealed an increase in the density of the wood in the RNAi lines compared to the wild type, although this was statistically significant only for line 3 (Fig. 4a). These young RNAi trees displayed slight differences in the total volume of the stem (Fig. 4b), which together with the changes in the density resulted in slight, but not statistically significant increase in the dry weight of the stem in lines 1 and 3 (Fig. 4c). Interestingly, trees that were grown for 6 months in the greenhouse developed a brown, striated bark (Fig. 4d) in contrast to the green and smooth bark of the wild-type trees of the same age (Fig. 4e). Anatomical inspection of the bark revealed that also the thickness of the suberized cork was significantly increased in all the three transgenic lines compared to the wild type (Fig. 4f-h).

**Fig. 5:**
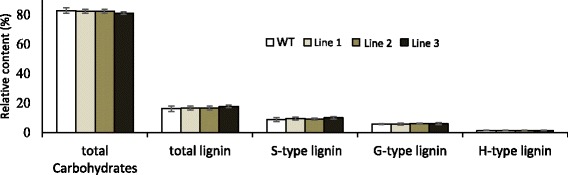
Pyrolysis gas chromatography/mass spectrometry (Py-GC/MS) analysis of the secondary xylem tissues. The relative content of carbohydrates and lignin is shown for mature xylem samples taken from the base of wild type and the *PttSCAMP3* RNAi trees. The relative content is calculated as the sum of the peak areas for the pyrolysis products derived from either the carbohydrate or lignin polymers, and are shown as a percentage of the total peak area from the GC-MS analysis. The composition of lignin is further shown as the relative content of the pyrolysis products derived from the S, G and H type lignin. *n* = 5

**Fig. 6:**
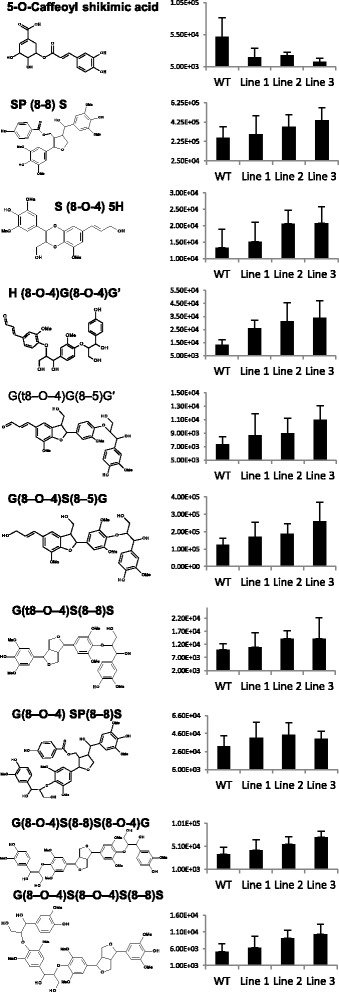
Small phenolic compounds accumulate in the secondary xylem of the *PttSCAMP3* RNAi lines. The graphs depict the abundance (peak area/mg fresh weight) and chemical structure of putative lignin-related oligomers in the *PttSCAMP3* RNAi lines and the wild type in the LC-MS metabolome analysis. Only metabolites having a |p(CORR)| ≥ 0.6 in the OnPLS analysis were included. Full data set for the LC-MS metabolome analysis can be found in Additional file 5. G, guaiacyl unit; S, syringyl unit; SP, unit derived from sinapyl p-hydroxybenzoate; H, *p*-hydroxyphenyl unit. *n* = 5

**Fig. 7:**
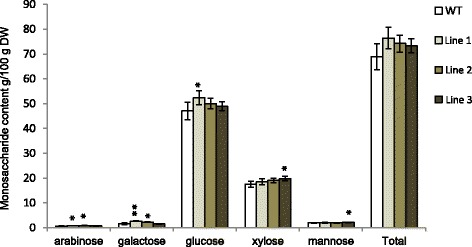
Monosaccharide yields in hydrolysates of the woody tissues of *PttSCAMP3* RNAi lines and wild type. Monosaccharide composition in wild type (WT) and *PttSCAMP3* RNAi lines detected after acid hydrolysis (72% *w*/w sulfuric acid). The hydrolysis releases arabinose, galactose, glucose, xylose and mannose from the secondary cell wall arabinan, galactan, glucan, xylan and mannan, respectively. Values are given as a percentage of g monosaccharide (in anhydrous form) per g dry weight of wood ± SD. Values are mean of three technical replicates, each of which containing equal amounts of wood powder pooled from five biological replicates for the transgenic lines and from five replicate pools of wild type trees. The “total” columns indicate the sum of the individual monosaccharide abundances. Asterisks indicate significant differences from the wild type at *P* < 0.05 (*) and *P* < 0.01 (**) according to Welch-corrected t-test

The expression pattern and changes in wood density prompted us to investigate the effect of *PttSCAMP3* RNAi expression on cell wall chemistry. A high-throughput analysis by pyrolysis gas chromatography/mass spectrometry (Py-GC/MS) did not reveal any significant differences in the relative content of carbohydrates and lignin even though a slight tendency towards higher lignin content was present especially in line 3 (Fig. 5). As Py-GC/MS reveals only the relative content of the cell wall components, alternative methods were used to identify possible differences in the absolute amounts of the cell wall components. An LC-MS metabolomic analysis revealed that the abundance of small phenolic compounds which were earlier identified as oligolignols [48] increased in abundance in the transgenic lines compared to the wild type (Fig. 6). The only compound that had a lower abundance in the transgenic lines was 5-O-caffeoyl shikimic acid which has been reported to inhibit activity of the lignin biosynthetic 4-coumaric acid:coenzyme A ligase (4CL) [49]. Detailed analysis of the carbohydrate composition by acid hydrolysis followed by HPAEC analysis also revealed increased abundance of monosaccharides derived from the major secondary cell wall carbohydrates glucan and xylan and from the minor carbohydrates arabinan and galactan in the RNAi lines compared to the wild type (Fig. 7). Taken together, the results support enhanced accumulation of both the carbohydrate and lignin components of the secondary cell walls in the woody tissues of the *PttSCAMP3* RNAi lines 1 and 3.

### Suppression of the *PttSCAMP* genes influences the bioprocessing properties of the wood

**Fig. 8:**
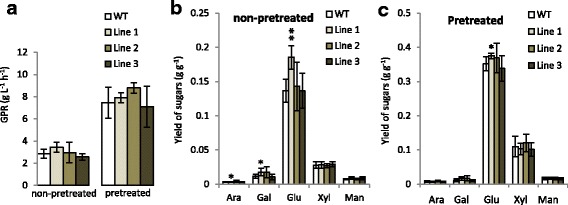
Susceptibility of the *PttSCAMP3* RNAi lines and wild type to enzymatic hydrolysis. **a** Glucose production rates (GPR) of wild type (WT) and *PttSCAMP3* RNAi lines after 2 h of enzymatic hydrolysis. The values represent means of GPR (g L^−1^ h^−1^ ± SD) in samples without (non-pretreated) and with an acid pretreatment (pretreated). **b** Sugar yields after enzymatic hydrolysis of non-pretreated woody tissues. The values represent amounts of the different monosaccharides (g monosaccharide per g dry weight) ± SD. **c** Sugar yields after enzymatic hydrolysis in pretreated woody tissues. The values represent combined sugar yields (g monosaccharide per g dry weight) ± SD from the pretreatment liquid and the enzymatic hydrolysate. Ara, arabinose; Gal, galactose, Glu, glucose; Xyl, xylose; Man, mannose. Asterisks indicate significant differences from the wild type at *P* < 0.05 (*) and *P* < 0.01 (**) according to a Welch-corrected t-test. *n* = 5

An increasingly important trait of forest trees is the susceptibility of the lignocellulosic raw material to enzymatic hydrolysis. To evaluate the effect of *PttSCAMP3* RNAi on this trait, an analytical scale pretreatment and enzymatic hydrolysis experiment was conducted for the wild type and the three different transgenic lines, and sugar yields were measured in woody material with and without an acid pretreatment. Interestingly, the lines behaved differently in these analyses. While line 1 showed a tendency towards increased glucose production rate (GPR; after 2 h of enzymatic hydrolysis) and significant increases in the yield of glucose both with and without the acid pretreatment, line 3 showed quite the opposite tendency towards decreased GPR and decreased yields of glucose and xylose that are the main sugars in the woody polymers (Fig. 8). The difference in the saccharification potential of the two lines might be due to the difference in the carbohydrate to lignin ratio of these lines; both lines showed increases in level of the carbohydrates (Fig. 7) that is expected to increase sugar yields after enzymatic hydrolysis, but this effect might be counteracted in line 3 by the increase in the relative content of lignin (Fig. 5) that is known to have an adverse effect on the saccharification potential.

### Integration of the multi-omics data by OnPLS modelling

To understand the mechanisms underlying the phenotypic changes in the *PttSCAMP3* RNAi lines, multi-omics analysis including transcriptomic, metabolomic and proteomic analysis of the transgenic lines and the wild type was performed in the living secondary xylem tissues from the same stem samples where the dead, mature tissues had been collected for the analyses of the wood chemistry and saccharification. To identify the most significant changes in relation to the phenotypic changes, OnPLS (orthogonal projections to latent structures) analysis was performed for data combined for the three transgenic lines from the five different omics platforms (transcriptomic, proteomic, GC-MS metabolomic, LC-MS metabolomic and Py-GC/MS metabolomic) and compared to the wild type. OnPLS [17, 18] is an extension of O2PLS [50, 51], and suitable for simultaneous analysis of more than two blocks of data or, as in our case, data from more than two platforms. It separates each data block into three parts: one where the data variation is globally joint (shared between all blocks or platforms in this case), one where the variation is locally joint (shared between some, but not all blocks/platforms) and one where the variation is unique to one block/platform. The analysis was introgressed into an OnPLS model, and the overview of the model was visualized by principal component analysis (Additional file 2A). The PCA analysis of the model revealed clear separation between the wild type and the transgenic *PttSCAMP3* lines. The majority of the modeled variation was either globally joint or locally joint; the sum of the global and location variation was 61% for the transcriptome, 70% for the proteome, 66% for the LC-MS metabolome, 73% for the GC-MS metabolome and 57% for the Py-GC/MS metabolome. A linear analysis of the five datablocks revealed the first component of each dataset as the main contributor accounting for the separation between the wild type and the transgenic lines (Additional file 2B). Statistical analysis was therefore performed on the basis of the first component. Large number of the variables were significantly different (|p(CORR)| > 0.5) between the transgenic lines and the wild type (Table 1; Additional files 3, 4, 5, 6 and 7). For both the proteome and the metabolome, the majority of the statistically significant changes resulted from increased rather than decreased abundance of the variables in the RNAi lines compared to the wild type (Table 1).

**Table 1:** Significantly different variables between the transgenic lines and the wild type in the different datasets on the basis of the OnPLS multivariate analysis

Dataset	Total number of variables	Number of the variables downregulated in transgenic lines compared to the wild type	Number of the variables upregulated in the transgenic lines compared to the wild type
Transcriptome	27,929	2639	2951
Proteome	1208	9	95
Metabolome (LC-MS)	1141	54	317
Metabolome (GC-MS)	214	12	22
Metabolome (Py-GC/MS)	109	17	38

The comparisons are done on the basis of the first component of the OnPLS analysis. |p(CORR)| > 0.5 was applied here as an arbitrary cutoff value to identify statistically significant variation between the transgenic lines and the wild type

### Multi-omics data provides clues to the function of the PttSCAMP proteins

Consistent with the expected function of the SCAMP proteins in membrane trafficking, a number of proteins that are known to be involved in secretion and/or endocytosis, such as secretion-associated RABA GTPase family protein Potri.016G000400 and a golgi snare protein Potri.014G066800, were significantly upregulated in the transgenic lines compared to the wild type (Table 2). Vice versa, three other RAB GTPase homologs (Potri.008G061300, Potri.003G081800, Potri.003G004100) were among the most downregulated proteins in the transgenic lines (Table 3).

**Table 2:** The most upregulated variables in the transgenic *PttSCAMP3* RNAi lines from the OnPLS analysis

Transcript		Protein		Metabolite (LC-MS)		Metabolite (GC-MS)	
Potri.010G017600 (receptor kinase 3)	−0.9	Potri.002G251600 (Actin binding Calponin homology (CH) domain-containing protein)	−0.8	324.0377@0.60600036	−0.9	Glycerol 1-palmitate	−0.8
Potri.008G125700 (RING-H2 finger A2A)	−0.9	Potri.006G168900 (SET domain-containing protein)	−0.8	Suberic acid	−0.9	Erythrose	−0.6
Potri.006G230700 (homeobox protein ATH1)	−0.9	Potri.015G108300	−0.8	396.1603@9.253999	−0.9	Myo-inositol-1-phosphate	−0.6
Potri.008G098300	−0.9	Potri.014G066800 (golgi snare 12)	−0.8	Monogalactosyldiacyl-glycerol (36:6)	−0.8	Gluconic acid-6-phosphate	−0.5
Potri.018G093100 (homolog of X-ray repair cross complementing 3	−0.9	Potri.018G060600	−0.8	888.5163@9.920005	−0.8	Oxalic acid	−0.5
Potri.011G081900	−0.9	Potri.019G053700 (isopentenyl-diphosphate delta-isomerase)	−0.8	Monogalactosyldiacyl-glycerol (36:6)	−0.8	Linoleic acid	−0.5
Potri.018G106700 (DNAse I-like superfamily protein)	−0.9	Potri.010G081600 (similar to auxin down-regulated protein ARG10)	−0.8	Monogalactosyldiacyl-glycerol (36:6)	−0.8	alpha-Linolenic acid	−0.5
Potri.009G166100	−0.9	Potri.012G074900 (B12D protein)	−0.8	Phosphatidic acid (36:6)	−0.8		
Potri.015G071100	−0.9	Potri.001G309200 (BolA-like family protein)	−0.8	458.0594@0.5979997	−0.8		
Potri.003G165800	−0.9	Potri.016G000400 (RAB GTPase homolog A2B)	−0.8	380.1651@9.253999	−0.8		
Potri.001G058600 (Squamosa promoter-binding protein-like)	−0.8	Potri.010G038400 (pyruvate dehydrogenase complex E1 α subunit)	−0.8	Hydroxycaproic acid	−0.8		
Potri.009G056200 (NB-ARC disease resistance protein)	−0.8	Potri.006G255800 (Myosin heavy chain-related protein)	−0.7	octadecadienoic acid	−0.8		
Potri.002G191900 (gibberellin 2-oxidase 6)	−0.8	Potri.006G107100 (aspartate aminotransferase 1)	−0.7	283.0899@1.0639995	−0.8		
Potri.003G063900 (ELF4-like 4)	−0.8	Potri.010G127500 (protein phosphatase 2A subunit A2)	−0.7	digalactosyldiacylglycerol (34:3)	−0.8		
Potri.003G100100 (Homeodomain-like protein)	−0.8	Potri.007G013400 (peptidylprolyl cis/trans isomerase)	−0.7	516.0613@0.6520003	−0.8		
Potri.016G110300 (Josephin family protein)	−0.8	Potri.008G217700 (cullin 1)	−0.7	digalactosyldiacylglycerol (34:3)	−0.8		
Potri.016G026600 (alpha-L-arabinofuranosidase 1)	−0.8	Potri.003G098400 (Proteasome component (PCI) domain protein)	−0.7	1118.559@9.75	−0.8		
Potri.002G065900 (5-FORMYLTETRAHYDRO-FOLATE CYCLO-LIGASE-RELATED)	−0.8	Potri.015G042600 (TCP-1/cpn60 chaperonin family protein)	−0.7	920.3991@11.602999	−0.8		
Potri.002G024300 (SAUR-like auxin-responsive protein family)	−0.8	Potri.018G091100 (Transducin/WD40 repeat-like superfamily protein)	−0.7	Digalactosyldiacyl-glycerol (36:6)	−0.8		
Potri.016G140200 (Leucine-rich repeat protein kinase)	−0.8	Potri.007G091000(Lipase/lipooxygenase, PLAT/LH2 family protein)	−0.7	934.5073@9.919004	−0.8		
Potri.014G129800 (complex 1 family protein/LVR family protein)	−0.8	Potri.012G069000 (METHYL-TRANSFERASE PMT2-RELATED)	−0.7	490.2831@10.1900015	−0.8		
Potri.003G111000	−0.8	Potri.016G087900 (adenylosuccinate synthase)	−0.7	5′-AMP	−0.8		
Potri.T128200 (Disease resistance protein family)	−0.8	Potri.001G206400 (Polymerase/ histidinol phosphatase-like)	−0.7	Phosphatidic acid (36:6)	−0.8		
Potri.T034300	−0.8	Potri.002G105100 (Clathrin adaptor complexes medium subunit family protein)	−0.7	830.4298@11.596002	−0.8		
Potri.005G148400 (AP2-LIKE ETHYLENE-RESPONSIVE TRANSCRIPTION FACTOR ANT	−0.8	Potri.T002400 (Disease resistance protein (TIR-NBS-LRR class) family)	−0.7	812.4986@9.919004	−0.8		
Potri.010G194400 (Yos1-like protein)	−0.8	Potri.001G021900	−0.7	737.4975@10.446007	−0.8		
Potri.017G059900 (ralf-like 32)	−0.8	Potri.006G138600 (chaperonin 20)	−0.7	124.0619@0.7949998	−0.8		
Potri.017G027800 (RHOMBOID-like protein 13)	−0.8	Potri.014G090100 (exocyst complex component sec10)	−0.7	453.9293@9.467004	−0.8		
Potri.016G073500	−0.8	Potri.004G140900 (cytochrome P450, family 707, subfamily A, polypeptide 4)	−0.7	Monogalactosyldiacyl-glycerol (36:6)	−0.8		
Potri.001G281600 (Lateral organ boundaries domain protein)	−0.8	Potri.013G128600 (ribosomal protein L5 B)	−0.7	689.1812@0.5330003	−0.8		

The variables are listed in descending order of statistical significance (p(CORR) from the OnPLS analysis. The p(CORR) value is depicted next to each variable. The transcript and protein annotations are according to JGI V3.0. LC-MS metabolites without annotations could not be identified and are listed as mass@retention time. Galactolipids can be present as adduct forms. Full datasets are listed in Additional files 3,4,5 and 6

**Table 3:** The most downregulated variables in the transgenic *PttSCAMP3* RNAi lines from the OnPLS analysis

Transcript		Protein		Metabolite (LC-MS)		Metabolite (GC-MS)	
Potri.002G066100 (Alba DNA/RNA-binding protein)	0.9	Potri.006G079700 (PREFOLDIN 1)	0.7	260.0366@7.6459966	0.8	Fructose-6-Phosphate	0.7
Potri.002G026600 (Regulator of chromosome condensation)	0.9	Potri.008G061300 (RAB GTPase homolog A2B)	0.6	170.0909@6.5109987	0.8	Succinic acid	0.6
Potri.008G014700 (calcium dependent protein kinase 1)	0.9	Potri.001G304700 (Ribosomal protein S5 domain 2-like superfamily protein)	0.6	‘UDP-galactose	0.8	Threonic acid	0.6
Potri.003G098200 (extra-large GTP-binding protein 3)	0.9	Potri.005G108100 (aconitase 1)	0.6	2-cis,4-trans-xanthoxin	0.7	Adenosine	0.5
Potri.018G044300	0.9	Potri.016G032500 (Single-stranded nucleic acid binding R3H protein)	0.6	312.2202@7.9389973	0.7	Malic acid	0.5
Potri.001G306700 (Protein of unknown function (DUF1278))	0.8	Potri.008G060000 (eukaryotic translation initiation factor 3G1)	0.6	iso-erythritol	0.7	Caffeic acid	0.5
Potri.001G428100 (NB-ARC disease resistance protein)	0.8	Potri.004G070000 (UDP-glucosyl transferase 88A1)	0.5	263.2075@8.526	0.7		
Potri.007G002400 (cytochrome P450, family 716, subfamily A,)	0.8	Potri.001G400900 (similar to cytidylyltransferase family)	0.5	86.041@0.7590002	0.7		
Potri.006G093800 (Inositol phosphorylceramide synthase 2)	0.8	Potri.001G464500 (germin-like protein 2)	0.5	280.1638@7.9479985	0.7		
Potri.005G181800 (Protein kinase superfamily protein)	0.8	Potri.019G050500 (Protein kinase superfamily protein)	0.5	438.3488@9.589997	0.7		
Potri.001G435700 (endoribo-nuclease L-PSP protein)	0.8	Potri.003G168500	0.5	157.0447@2.569	0.7		
Potri.015G079500 (scramblase-related)	0.8	Potri.013G062500	0.5	5-O-Caffeoylshikimic acid	0.7		
Potri.011G100900 (ARF-GAP domain 13)	0.8	Potri.015G090900 (26S proteasome, regulatory subunit Rpn7)	0.5	392.0447@0.6760002	0.6		
Potri.006G187500 (Calcineurin-like metallo-phosphoesterase)	0.8	Potri.009G120500 (regulatory particle triple-A ATPase 4A)	0.5	98.0651@4.285999	0.6		
Potri.014G148800 (DNA topoisomerase, type IA, core)	0.8	Potri.010G069900 (Ribosomal protein L14)	0.5	404.0859@3.440001	0.6		
Potri.016G018700	0.8	Potri.011G110900 (general regulatory factor 9)	0.5	157.0442@2.1259997	0.6		
Potri.006G085400 (aminoacyl-tRNA and biotin synthetase)	0.8	Potri.006G073200 (Ribosomal protein L30/L7 family protein)	0.5	243.1821@6.7790008	0.6		
Potri.001G420400 (SMAD/FHA domain-containing protein)	0.8	Potri.012G062600 (ribulose-bisphosphate carboxylase)	0.4	310.1765@7.268003	0.6		
Potri.017G082800	0.8	Potri.002G082101	0.4	103.0391@0.7649997	0.6		
Potri.011G169200	0.8	Potri.002G057300 (Pleckstrin homology (PH) domain-containing protein)	0.4	422.2356@9.213004	0.6		
Potri.003G098100 (GHMP kinase family protein)	0.8	Potri.002G182500 (CARBON CATABOLITE REPRESSOR PROTEIN 4)	0.4	537.1221@1.2070005	0.6		
Potri.002G006700 (MLO family protein)	0.8	Potri.008G012400 (FASCICLIN-like arabinogalactan protein 17 precursor)	0.4	537.1187@1.2060003	0.6		
Potri.008G159700 (Pyruvate kinase family protein)	0.8	Potri.018G078200 (ankyrin repeat family protein)	0.4	Succinic acid	0.6		
Potri.004G106200 (phosphoglycerate mutase)	0.8	Potri.002G045700 (tryptophan synthase alpha chain)	0.4	148.0429@0.859	0.6		
Potri.011G167000 (amino acid permease 7)	0.8	Potri.006G275000 (H(+)-ATPase 5)	0.4	464.1305@4.291	0.6		
Potri.001G004600 (tubulin α-3)	0.8	Potri.003G081800 (RAS 5)	0.4	464.1145@4.291	0.6		
Potri.002G171800 (cytochrome P450, family 703, subfamily A)	0.8	Potri.001G194000 (Ribosomal L28e protein family)	0.4	7-Hydroxyflavone	0.6		
Potri.005G028900 (Rab escort prot)	0.8	Potri.003G004100 (RAB GTPase 11C)	0.4	305.9969@0.57099974	0.6		
Potri.T060400 (NB-ARC disease resistance protein)	0.8	Potri.T106200 (Peroxidase superfamily protein)	0.4	252.0948@6.714998	0.6		
Potri.001G132900 (ENHANCED DISEASE RESISTANCE 2)	0.8	Potri.007G014300 (Histone superfamily protein)	0.4	419.9017@0.41500008	0.6		

The variables are listed in descending order of statistical significance (p(CORR) from the OnPLS analysis. The p(CORR) value is depicted next to each variable. The transcript and protein annotations are according to JGI V3.0. LC-MS metabolites without annotations could not be identified and are listed as mass@retention time. Full datasets are listed in Additional files 3,4,5 and 6

One of the most striking changes in the *PttSCAMP3* RNAi lines concerned increased abundance of lipids, such as linolenic acid, linoleic acid and glycerol-1-palmitate (Table 2; Additional file 6). Also several galactolipids such as monogalactosyldiacylglycerol (MGDG) and diagalactosyldiacylglycerol (DGDG) as well as their precursor phosphatidic acid (PA) were increased in abundance (Additional file 8). Since galactolipids are known to be localized in the chloroplast membranes, their localization in the differentiating xylem elements is most probably in the parenchymatic ray cells that are the only chloroplast-containing cells of the xylem.

### Multi-omic analysis of the cell wall biosynthetic pathways

**Fig. 9:**
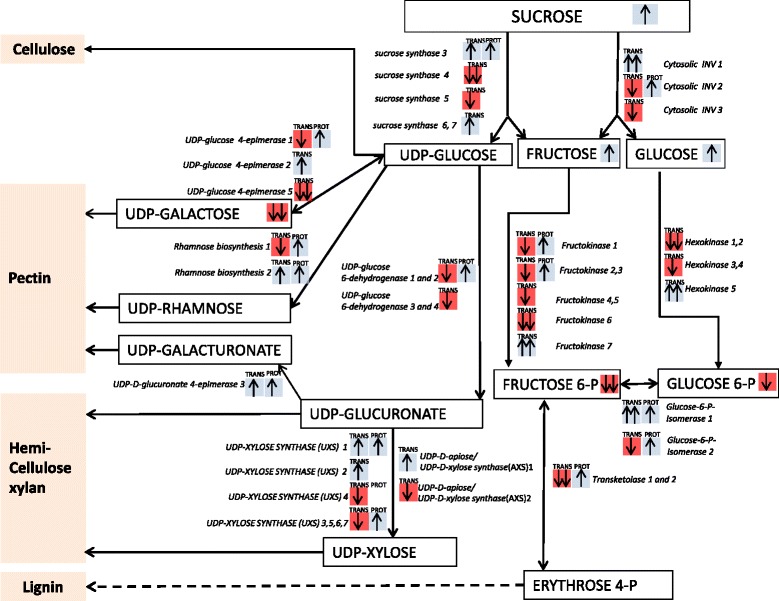
Analysis of the cell wall biosynthetic pathway on the transcript, protein and metabolite levels. The abundance of the secondary cell wall-related metabolites, transcripts (Trans) and proteins (Prot) are shown in the *PttSCAMP3* RNAi lines compared to the wild type. ↑ in the blue boxes indicates upregulation in *PttSCAMP3* RNAi lines; ↑↑ in the blue boxes indicates significant (|p(CORR)| >0.5) upregulation in *PttSCAMP3* RNAi lines; ↓ in the red boxes indicates downregulation in the *PttSCAMP3* RNAi lines; ↓↓ in the red boxes indicates significant (|p(CORR)| > 0.5) downregulation in *PttSCAMP3* RNAi lines. Metabolites are shown in bold in boxes with the arrow/color codes as indicated above. The genes and proteins correspond to the following *Populus trichocarpa* gene models according to JGI V3.0: Fructokinase 1 (Potri.017G126300); Fructokinase 2 (Potri.007G129700); Fructokinase 3 (Potri.017G029000); Fructokinase 4 (Potri.012G132700); Fructokinase 5 (Potri.004G089300); Fructokinase 6 (Potri.015G134900); Fructokinase 7 (Potri.019G063600); Hexokinase 1 (Potri.001G190400); Hexokinase 2 (Potri.005G238600); Hexokinase 3 (Potri.009G050000); Hexokinase 4 (Potri.018G088300); Hexokinase 5 (Potri.001G254800); Cytosolic INV 1 (Potri.014G188100); Cytosolic INV 2 (Potri.013G110800); Cytosolic INV3 (Potri.019G082000); sucrose synthase 3 (Potri.002G202300); sucrose synthase 4 (Potri.006G136700); sucrose synthase 5 (Potri.018G063500); sucrose synthase 6 (Potri.004G081300); sucrose synthase 7 (Potri.012G037200); glucose-6-phosphate isomerase 1 (Potri.008G118900); glucose-6-phosphate isomerase 2 (Potri.002G104000); UDP-D-apiose/UDP-D-xylose synthase 1 (Potri.009G150600); UDP-D-apiose/UDP-D-xylose synthase 2 (Potri.004G189900); UDP-glucose 6-dehydrogenase 1 (Potri.017G092000); UDP-glucose 6-dehydrogenase 2 (Potri.004G118600); UDP-glucose 6-dehydrogenase 3 (Potri.010G159800); UDP-glucose 6-dehydrogenase 4 (Potri.008G094300); UDP-glucose 4-epimerase 1 (Potri.003G123700); UDP-glucose 4-epimerase 2 (Potri.003G140900); UDP-glucose 4-epimerase 5 (Potri.001G090700); UDP-D-xylose synthase (UXS) 1 (Potri.006G214000); UDP-D-xylose synthase (UXS) 2 (Potri.014G129200); UDP-D-xylose synthase (UXS) 3 (Potri.010G207200); UDP-D-xylose synthase (UXS) 4 (Potri.002G204400); UDP-D-xylose synthase (UXS) 5 (Potri.001G237200); UDP-D-xylose synthase (UXS) 6 (Potri.008G053100); UDP-D-xylose synthase (UXS) 7 (Potri.016G080500); Transketolase 1 (Potri.002G146300); Transketolase 2 (Potri.014G068200); UDP-D-glucuronate 4-epimerase (Potri.002G146500); Rhamnose biosynthesis 1 (Potri.006G272700); Rhamnose biosynthesis 2 (Potri.001G383500). Observe that the numbering of the genes is arbitrary to allow identification of the different members of the gene family

The multi-omic analyses revealed numerous differences in carbon metabolism and cell wall biosynthesis of the *PttSCAMP3* RNAi lines. A detailed analysis revealed increased abundance of sucrose, fructose and glucose in the transgenic lines compared to the wild type (Additional file 6; Fig. 9). In addition, enzymes catalyzing cell wall monomer biosynthesis were frequently more abundant in the transgenic lines. Both a sucrose synthase (SuSy3) and a cytosolic invertase which produce nucleotide sugars and neutral fructose and glucose for biosynthesis of various cell wall components, were more abundant in the transgenic lines compared to the wild type (Additional file 4; Fig. 9). Also enzymes that produce monomers for secondary cell wall hemicellulose (xylan) biosynthesis (UDP-glucose 6-dehydrogenase and UDP-xylose synthase) were more abundant on a protein level, but mostly suppressed on the transcript level in the transgenic lines compared to the wild type (Fig. 9). Also enzymes that are responsible for producing precursors for pectin in the primary cell walls were mostly more abundant in the transgenic lines compared to the wild type (Fig. 9). Overall, the enzymes corresponding to cell wall biosynthesis were more abundant on the protein level but suppressed on the transcriptional level.

## Discussion

The plant SCAMP proteins are believed to function in various membrane trafficking pathways on the basis of structural conservation with the animal systems as well the plant localization studies, but no functional evidence has been reported earlier most probably due to functional redundancy of the gene family [19]. Our study in *Populus* trees identified a role for plant SCAMPs in wood formation. The *PttSCAMP3* RNAi lines exhibited increased deposition of both the carbohydrate and the phenolic components of the woody tissues of the stem, suggesting function of the studied *SCAMP* genes in suppressing deposition of the secondary cell wall components. In spite of the changes in cell wall polymers no growth penalties were observed in the transgenic lines, which demonstrates the potential of *SCAMP3* suppression in improving biomass properties in forest trees.

The different RNAi lines differed somewhat in their phenotypes. Line 1 showed increased content of the glucan polysaccharide (Fig. 7), which correlated with increased glucose yields after enzymatic saccharification of this line (Fig. 8). Line 3 showed slightly increased lignin content and increased accumulation of xylan which is the main secondary cell wall hemicellulose (Figs. 6 and 7). Both xylan and lignin are known to act negatively on enzymatic hydrolysis of the secondary cell walls, possibly then explaining the absence of increased sugar yields in the saccharification assays of the woody tissues in line 3. These phenotypic differences could depend on the effect of the RNAi construct in suppressing only *PttSCAMP6* in line 1 and both *PttSCAMP3* and *PttSCAMP6* in line 3 (Fig. 3a), and hence slightly different roles of these two *Populus* SCAMP proteins in membrane trafficking. Differential action of *PttSCAMP3* and *PttSCAMP6* is supported by the slightly different expression patterns in the differentiating xylem tissues of the stem (Fig. 2). The two earlier characterized plant SCAMP proteins, *Os*SCAMP1 in rice and *Nt*SCAMP2, were also shown to have partially differential localization along the secretory pathway and hence most probably at least partially different functions. While OsSCAMP1 was localized to the plasmamembrane, trans-golgi network and cell plate [52], *Nt*SCAMP2 was in addition found on the secretory vesicle cluster [46].

Overaccumulation of the cell wall components can be achieved by increased secretion of these compounds and/or increased trafficking of proteins, such as cell wall monomer biosynthetic enzymes or transporters, that are critical for cell wall deposition. Increased secretion would explain increased accumulation of xylan that is synthesized in the golgi and secreted to the cell wall, whereas increased trafficking of cellulose biosynthetic CesA enzymes would in turn enhance accumulation of cellulose. Overaccumulation of the lignin precursors is more difficult to explain since the transport mechanisms of lignin monomers are not fully understood even though ABC transporters are believed to be involved [53–55]. Four ABC transporter proteins were identified in the *PttSCAMP3* transgenic lines (Potri.002G036400, Potri.010G003000, Potri.014G113200 and Potri.015G023800). Two of these (Potri.010G003000 and Potri.015G023800) were more abundant in the transgenic lines compared to the wild type. According to the proposed function of the *Arabidopsis* homolog, Potri.010G003000 is involved in auxin efflux. The function of Potri.015G023800 is not known and it is possible that this ABC transporter could be involved in transport of monomers for lignin.

Another mechanism to control accumulation of secreted compounds and proteins is to affect the secretory machinery itself. The tobacco *Nt*SCAMP2 was localized to the secretory vesicle clusters which are responsible for mass secretion to the cell wall [46], and it is possible that the *Populus* SCAMP proteins control some function of such vesicles. It was interesting that *PttSCAMP3* was highly co-expressed with nucleotide-diphospho-sugar transferases (Additional file 1). Accordingly, a nucleotide-diphospho-sugar transferase (Potri.001G400900) was significantly suppressed in abundance in the transgenic RNAi lines (Table 3), which supports the role of the PttSCAMPs in control of this kind of proteins. Yet another indication in this direction is provided by DeBolt et al. [56] who showed that a mutation in a dinucleotide sugar transferase UGT80B1 decreased suberin accumulation in *Arabidopsis,* and proposed that UGT80B1 glycosylates sterols that control trafficking of lipid precursors for instance for suberin biosynthesis. It is therefore possible that the PttSCAMPs are crucial for the function of some dinucleotide sugar transferases that, like UGT80B1, affect membrane trafficking and hence secretion of cell wall components and/or biosynthetic enzymes. According to this scenario, the PttSCAMPs would function as a safeguard that normally suppress secretion of cell wall precursors. Changes in the PttSCAMP-mediated trafficking would then allow rapid modification in the level of the cell wall polymers whenever necessary.

## Conclusions

In the current study, we demonstrated a function for *Populus* SCAMP proteins in deposition of cell wall components in woody tissues of *Populus* trees. Even though only small differences were evident in comparisons using traditional statistical methods, the OnPLS model provided clear separation between the transgenic lines and the wild type. This demonstrates the strength of OnPLS modeling in handling simultaneously very different kinds of datasets and the intrinsic property of the model to readily identify small but consistent variation between the different datasets. The modelling also allowed identification of possible mechanisms underlying the phenotypic changes in the *PttSCAMP3* transgenic trees and hence putative functions for the *Populus* SCAMP genes. These datasets provide a solid basis for understanding and further exploration of this poorly understood gene family in plants.

## Additional files

Additional file 1: Coexpression analysis of *PttSCAMP3* in AspWood. Closest neighbours of *PttSCAMP3* were identified in AspWood database (http://aspwood.popgenie.org/aspwood-v3.0/) using the default settings. (XLSX 21 kb)

Additional file 2: The multivariate modelling of the data. **A**. PCA scores overview of the OnPLS model for the wild type (WT) and transgenic *PttSCAMP3* RNAi lines. PCA is based on all variation (global, local and unique variation) in the OnPLS model. **B**. Contribution of the PCA loadings for the different datasets to the overall variation between the transgenic lines and the wild type in the OnPLS model. The datasets are the following: LCMS, LC-MS metabolomic dataset; Trans; transcriptomic dataset; Prot, proteomic dataset; GC, GC-MS metabolomic dataset; MSPy, Pyrolysis-gas chromatography/mass spectrometry dataset. The numbers 1–7 refer to the number of PCA component for each particular dataset. (PPTX 126 kb)

Additional file 3: RNAseq analysis of wild type and *PttSCAMP3* RNAi plants. (XLSX 1841 kb)

Additional file 4: Proteomic analysis of wild type and *PttSCAMP3* RNAi plants. **4.1.** List of all peptides identified in the proteomic study. Statistical (OnPLS) analysis was done at the peptide level to compare the data from the wild type and the combined data from the three transgenic lines. The statistical analysis on the protein level is based on the p(CORR) values of the cognate peptides. **4.2**. List of all proteins identified in the proteomic study on the basis of the cognate unique peptides. The p(CORR) value is a median for the p(CORR) values of the unique peptides corresponding to each of the proteins, and depict statistical significance between the wild type and the transgenic lines. (XLSX 698 kb)

Additional file 5: LC-MS metabolomic analysis of wild type and *PttSCAMP3* RNAi plants. (XLSX 64 kb)

Additional file 6: GC-MS metabolomic analysis of wild type and *PttSCAMP3* RNAi plants. (XLSX 19 kb)

Additional file 7: Py-GC/MS analysis of wild type and *PttSCAMP3* RNAi plants. (XLSX 14 kb)

Additional file 8: LC-MS metabolomic identification of lipids. The graphs depict the abundance of lipids (peak area/mg fresh weight) in the *PttSCAMP3* RNAi lines compared to the wild type in the LC-MS metabolome analysis. Only metabolites having |p(CORR)| ≥ 0.6 were included. The full LC-MS metabolome dataset is listed in Additional file 5. DGDG, digalactosyldiacylglycerol; MGDG, monogalactosyldiacylglycerol; PA, phosphatidic acid. (PPTX 1263 kb)

## Abbreviations

DGDG: Digalactosyldiacylglycerol
GC-MS: Gas chromatography-mass spectrometry
GPR: Glucose production rate
HPAEC: High-performance anion-exchange chromatography
LC-MS: Liquid chromatograph-mass spectrometry
MGDG: Monogalactosyldiacylglycerol
OnPLS: Orthogonal projections to latent structures
PA: Phosphatidic acid
PCA: Principal component analysis
Py-GC/MS: Pyrolysis gas chromatography/mass spectrometry
RNAi: RNA interference
SCAMP: Secretory Carrier-Associated Membrane Proteins
VST: Variance-stabilizing transformation
WT: Wild type
