# Supplementary figures and images for: A multi-omics approach reveals function of Secretory Carrier-Associated Membrane Proteins in wood formation of​ ​​Populus​​ ​trees
Source: BMC Genomics. 2018 Jan 3;19:11. doi: 10.1186/s12864-017-4411-1 (PMC5753437; doi:10.1186/s12864-017-4411-1)

## Slide 1
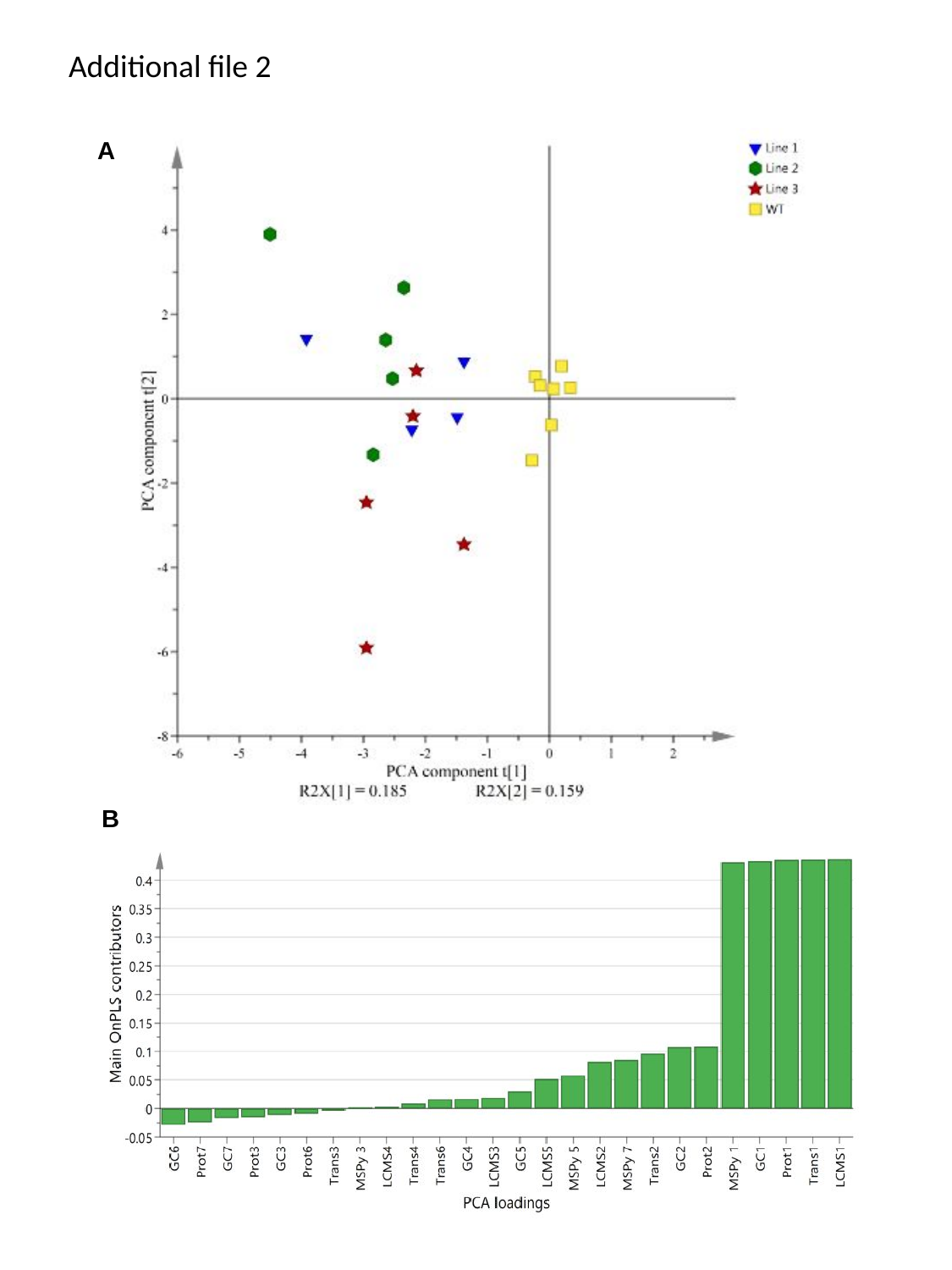

Additional file 2
A
B

Supplement: Supplementary file 2 — The multivariate modelling of the data. A. PCA scores overview of the OnPLS model for the wild type (WT) and transgenic PttSCAMP3 RNAi lines. PCA is based on all variation (global, local and unique variation) in the OnPLS model. B. Contribution of the PCA loadings for the different datasets to the overall variation between the transgenic lines and the wild type in the OnPLS model. The datasets are the following: LCMS, LC-MS metabolomic dataset; Trans; transcriptomic dataset; Prot, proteomic dataset; GC, GC-MS metabolomic dataset; MSPy, Pyrolysis-gas chromatography/mass spectrometry dataset. The numbers 1–7 refer to the number of PCA component for each particular dataset. (PPTX 126 kb) [file 12864_2017_4411_MOESM2_ESM.pptx]
