# Supplementary material for: A multi-omics approach reveals function of Secretory Carrier-Associated Membrane Proteins in wood formation of​ ​​Populus​​ ​trees
Source: BMC Genomics. 2018 Jan 3;19:11. doi: 10.1186/s12864-017-4411-1 (PMC5753437; doi:10.1186/s12864-017-4411-1)
Supplement: Supplementary file 8 — LC-MS metabolomic identification of lipids. The graphs depict the abundance of lipids (peak area/mg fresh weight) in the PttSCAMP3 RNAi lines compared to the wild type in the LC-MS metabolome analysis. Only metabolites having |p(CORR)| ≥ 0.6 were included. The full LC-MS metabolome dataset is listed in Additional file 5. DGDG, digalactosyldiacylglycerol; MGDG, monogalactosyldiacylglycerol; PA, phosphatidic acid. (PPTX 1263 kb) [file 12864_2017_4411_MOESM8_ESM.pptx]

## Slide 1
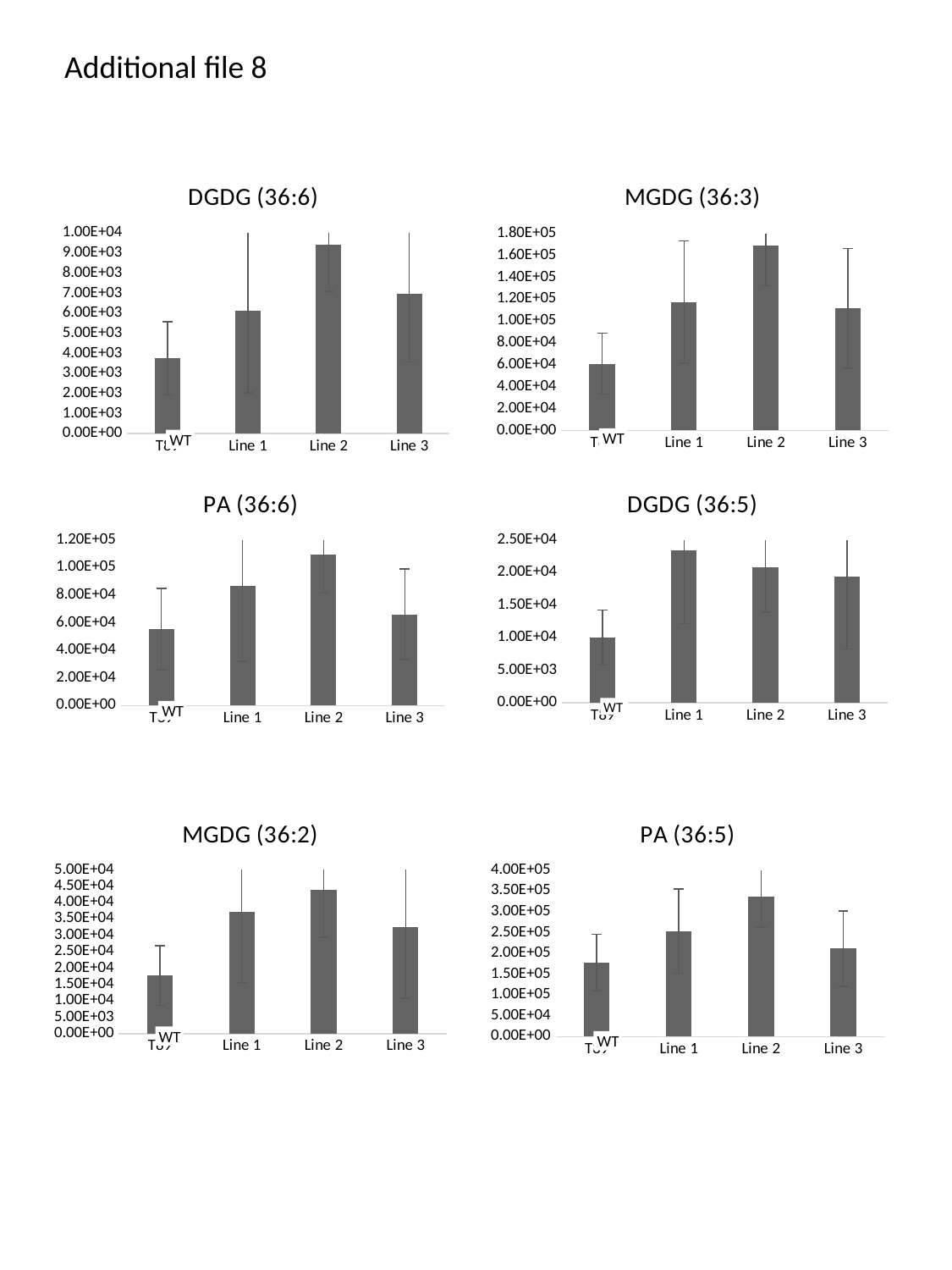

Additional file 8
### Chart: DGDG (36:6)
| Category | |
|---|---|
| T89 | 3748.5714285714284 |
| Line 1 | 6123.5 |
| Line 2 | 9403.6 |
| Line 3 | 6984.2 |
### Chart: MGDG (36:3)
| Category | |
|---|---|
| T89 | 61045.57142857143 |
| Line 1 | 117414.75 |
| Line 2 | 168607.4 |
| Line 3 | 111660.2 | WT
 WT
### Chart: DGDG (36:5)
| Category | |
|---|---|
| T89 | 10004.857142857143 |
| Line 1 | 23427.5 |
| Line 2 | 20770.6 |
| Line 3 | 19314.6 |
### Chart: PA (36:6)
| Category | |
|---|---|
| T89 | 55282.42857142857 |
| Line 1 | 86662.75 |
| Line 2 | 109660.4 |
| Line 3 | 66017.2 | WT
 WT
### Chart: PA (36:5)
| Category | |
|---|---|
| T89 | 177942.14285714287 |
| Line 1 | 253148.5 |
| Line 2 | 335743.8 |
| Line 3 | 211056.6 |
### Chart: MGDG (36:2)
| Category | |
|---|---|
| T89 | 17670.428571428572 |
| Line 1 | 37244.75 |
| Line 2 | 43838.6 |
| Line 3 | 32457.8 | WT
 WT
